# Reconstruction of Dynamic and Reversible Color Change using Reflectin Protein

**DOI:** 10.1038/s41598-019-41638-8

**Published:** 2019-03-26

**Authors:** Tiantian Cai, Kui Han, Peilin Yang, Zhou Zhu, Mengcheng Jiang, Yanyi Huang, Can Xie

**Affiliations:** 1 State Key Laboratory of Membrane Biology, Laboratory of Molecular Biophysics, School of Life Sciences, Peking University, Beijing, 100871 China; 2 Biodynamic Optical Imaging Center (BIOPIC), Beijing Advanced Innovation Center for Genomics (ICG), Peking-Tsinghua Center for Life Sciences, College of Engineering, and School of Life Sciences, Peking University, Beijing, China; 3 Beijing Computational Science Research Center, The Chinese Academy of Engineering Physics, Beijing, 100084 China

## Abstract

Cephalopods have remarkable ability to change their body color across a wide range of wavelengths, yet the structural basis remains largely unknown. Reflectin, a protein family assumed to be responsible for structural color in cephalopods, has unique features of higher-order assembly that are tightly regulated by aromatic molecules. Here, we reconstructed the dynamic and reversible color change using purified reflectin protein and demonstrated how the conformational change and the status of assembly led to the change in optical properties. In addition, optical spectral and structural analyses indicated that the “cephalopod-blue” primarily resulted from wavelength-dependent light scattering rather than reflection. Our results suggest a possible role of reflectin in color dynamics. The *in vitro* reconstruction system we present here may serve as an initial step for designing bio-inspired optical materials based on reflectin protein.

## Introduction

Colors in nature are primarily mediated by pigmentary or structural elements or a combination of both^1–3^. In contrast to pigment-derived colors, structural colors are produced by the physical interactions of the incident light with micro- or nano-structures^1,4^. Structural colors are bright, often iridescent, and widely used by living organisms for camouflaging or signaling^5^. Blue pigments are rare with limited reports in the animal kingdom, such as blue crustacyanin in lobsters^6,7^ and blue-green bile pigments in butterflies^8^. Therefore, structural colorations contribute to most of brilliant observed in some butterflies^9^, birds^3,10^, and mammals^2^ and even in the dynamic color change in cephalopods^11^, which represent one of the most unique and sophisticated coloration systems in nature.

Cephalopods, the animal group including octopus, cuttlefish and squid, are masters at manipulating their body coloration and patterns for camouflage or intra-species communication^12,13^. This remarkable ability of cephalopods depends on their unique skin structure containing multiple types of cells located in different layers with different functions^14^. Pigment-containing sacs in chromatophores located in the dermal layer are innervated and partially responsible for color change over the range of a few seconds^15^. Structural color change mediated by reflectin-formed insoluble platelets in iridophores or spheres in leucophores beneath chromatophores requires a longer amount of time^1^.

Reflectin, a protein family named because of its high-refractive index, is expressed exclusively in cephalopods^16^. The photonic properties of reflectin have attracted considerable attention and intensive investigations for a long time. The structural colors in cephalopods are suggested by multi-layer interference when incident light reflected by periodically layered reflectin platelets in iridophores^17,18^, and several experimental validations have been carried out at protein^19,20^ and cellular levels^18^, however, the structural basis of the color change process remains unknown. Reflectin also exhibits multifaceted features both *in vivo* and *in vitro*^1,16,17,20–27^. It forms ribbon-shaped, cup-shaped and discoidal platelets in iridophores of different species^14^; spheres with various diameters in leucophores^23^ and chromatophores *in vivo*^28^; and either globular “reflectin bricks” from self-assembly or huge platelet structures with different shapes from higher order assembly *in vitro*^26^. The plasticity and the multifaceted features of platelet “Bragg reflector” in cephalopods can be traced to the special amino acid composition in protein sequence^16^, extremely low solubility in biochemistry^16,26^, and remarkable assembly ability of the reflectin protein structure^17,20,25,26,29^. However, the connection between the complexity of structural color change in cephalopods and the structural/assembly status change of reflectin protein remains unresolved.

One method to circumvent the question is to reconstruct the dynamic color change process with purified reflectin protein *in vitro*, thereby reducing the complexity of biological processes into simple molecular interactions, thus the structural/assembly status and optical features can be monitored at the same time. Here, reflectin protein was spin coated to form thin solid films to mimic the platelet structures in cephalopod iridophores. Using this clean-cut approach, the dynamic structural color change process from colorless to white and blue was successfully regenerated *in vitro*. We demonstrated that the blue coloration (here named “cephalopod-blue”) was only produced by higher-order assembly of reflectin proteins in the presence of aromatic triggers. Reflection and transmission spectra indicate that “cephalopod-blue” is a result of wavelength-dependent light scattering, as confirmed by structural analysis of the reflectin films. The results outlined here may facilitate further design of bio-inspired photonic materials, and may provide biological implications to our understanding of the structural color change in cephalopods.

## Results

### Coloration of reflectin

**Figure 1:**
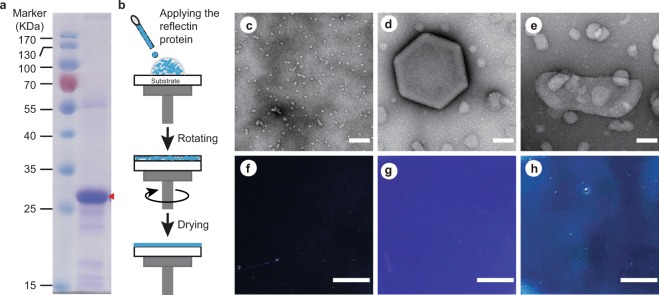
Film formation and coloration of Reflectin. (**a**), SDS-PAGE of purified Reflectin (SoRef2). (**b**) Schematic of the spin-coating process. (**c**) Negative-staining EM image of Reflectin (SoRef2) particles with no aromatic molecules. (**d,e**) Negative-staining EM image of Reflectin (SoRef2) incubated with imidazole (Reflectin/Imidazole) (**d**) and histamine (Reflectin/Histamine) (**e**). (**f**) Film generated by spin coating the Reflectin sample in (**c**). (**g**) Film generated by spin coating the Reflectin/Imidazole sample in (**d**). (**h**) Film generated by spin coating the Reflectin/Histamine sample in (**e**). The scale bars in (**c**–**e**) represent 100 nm, whereas the scale bars in (**f**–**h**) represent 2 mm.

Recombinant reflectin proteins have been applied in numerous studies to fabricate reflectin films to elucidate the origin of reflectin-based structural colors^17,19,20,30,31^. In these studies, reflectin proteins were dissolved in organic solvents, such as hexafluoroisopropanol (HFIP) or trifluoroacetic acid (TFA), followed by flow coating or spin coating. Thin-film interference was suggested as one possible mechanism of coloration. With multiple reflectin proteins purified in mild buffer solution (represented by SoRef2 from *Sepia officinalis*, EsRef1a from *Euprymna scolopes* and DpRefA2 from *Doryteuthis pealeii*), we previously demonstrated tightly regulated hierarchical assembly of reflectin, and this ability was even preserved by its single domain^26^. To explore how the assembly status of reflectin may affect structural coloration, we revisited the structure-optics relationship by spin-coated reflectin at different assembly stages. One reflectin, SoRef2 (GenBank: HE687200.1), was expressed and purified to homogeneity (Fig. 1a), incubated with or without aromatic reagents (imidazole and histamine), and then spin coated as described^19^ (Fig. 1b). In contrast to reflectin proteins in organic solvents, reflectin proteins were added to buffer solution to maintain their conformation. Transmission electron microscopes (TEM) were used to monitor reflectin conformation and assembly status with various reflectin/reagents ratios (Fig. S1). We observed hierarchical assembly as reported previously^26^. Both small globular particles (Fig. 1c) from self-assembly and huge platelet structures (Fig. 1d,e) from higher-order assembly in different reflectin samples in the absence or presence of aromatic stimulus were observed, respectively. To our surprise, the appearance color of the film depended on the treatment of reflectin protein. Film formed by self-assembled reflectin without aromatic molecules remains colorless (Fig. 1f), whereas films formed by higher-order assembled reflectin protein incubated with imidazole (Fig. 1g) and histamine (Fig. 1h) exhibit similar blue color.

Multiple reflectin genes exist in cephalopods, and different reflectin proteins exhibit specific distributions *in vivo*, which may suggest different roles in the structural color change process^32^. To investigate whether different reflectin proteins are responsible for different optical features, two more representative reflectin proteins from *Sepia officinalis* (SoRef1, GenBank: HE687199.1 and SoRef8, GenBank: HE687206.1) were selected based on sequence similarity analysis (Fig. S2a,b), expressed and purified (Fig. S2c,h). Similar structures and assembly features compared with SoRef2 were confirmed by TEM with or without aromatic molecules (Fig. S2d,e for SoRef1; Fig. S2i,j for SoRef8). The similar blue coloration was obtained for higher ordered assembled SoRef1 (Fig. S2f,g) and SoRef8 (Fig. S2k,l) in the presence of aromatic molecules.

The assembly capacity of reflectin is well preserved in its single domain^26^. To further explore whether one single domain of reflectin is sufficient to produce the similar optical appearances, a single domain (D1) of SoRef2 was obtained (Fig. S2m–q). The characteristic blue was also observed in the film formed by D1 protein in the presence of aromatic triggers (Fig. S2q). Thus, we concluded that the blue coloration was related to the higher order assembled structures of reflectin protein, and this optical feature was largely preserved even in its single domain.

### Optical characterizations of reflectin films

**Figure 2:**
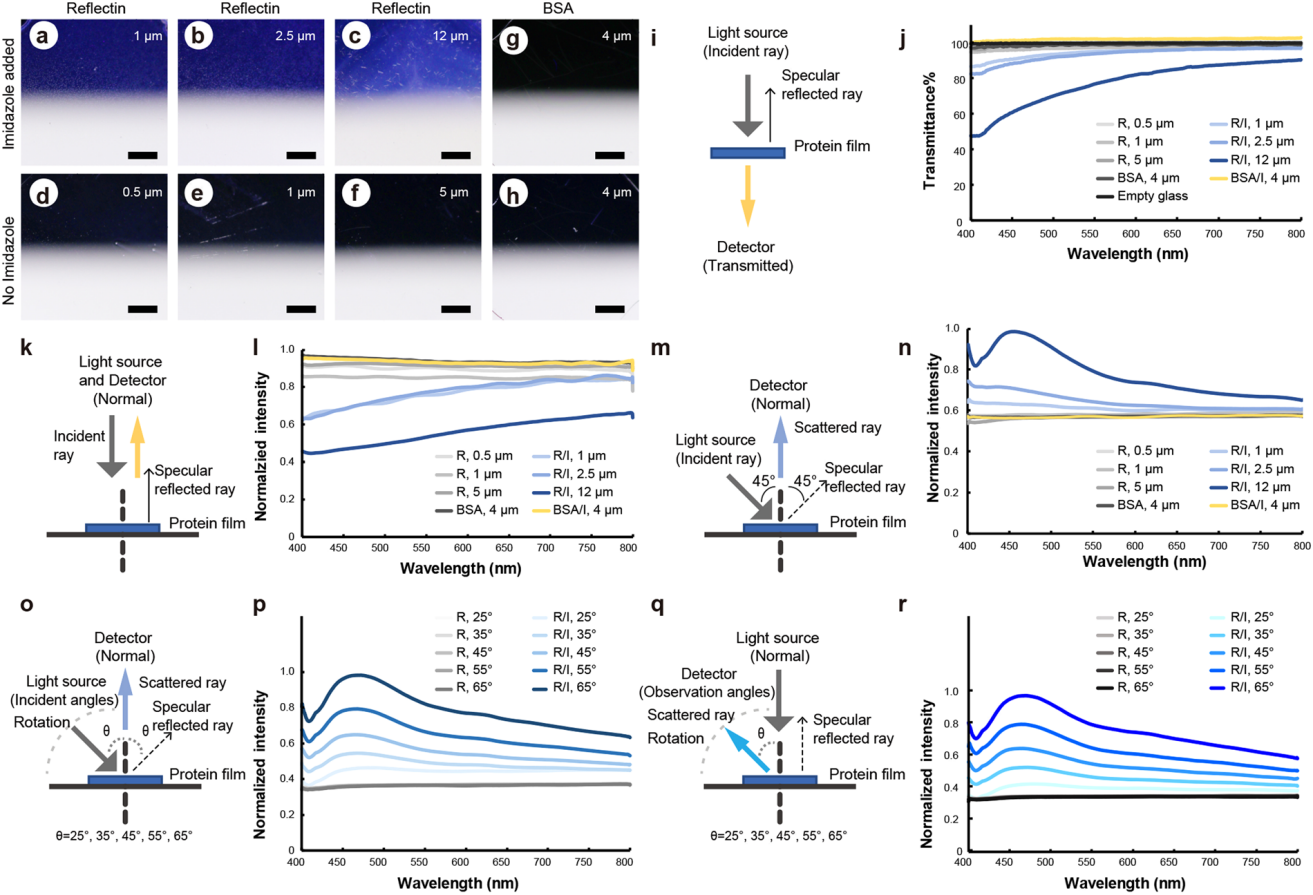
Optical characterization of Reflectin films. (**a**–**f**) Films with various thicknesses (1 µm (**a**), 2.5 µm (**b**), 12 µm (**c**), 0.5 µm (**d**), 1 µm (**e**) and 5 µm **(f**)) generated by spin coating of Reflectin (SoRef2) incubated with and without imidazole on glasses, labeled as R/I (Reflectin/Imidazole) and R (Reflectin), respectively. All the pictures were obtained against black background (upper half) with a white background (lower half) underneath. Reflectin with imidazole (R/I) films appear blue on a black background and light yellow on a white background, respectively (**a**–**c**). In contrast, reflectin films without imidazole (R) are colorless (**d**–**f**). (**g**,**h**) Spin-coated films (4 µm) of BSA incubated with (**g)** and without (**h)** imidazole, labeled as BSA/I and BSA, respectively. Both films are colorless. **i**, Schematic of setup for transmittance measurement used in this work. (**j**) Transmittance spectra of Reflectin and BSA films in (**a**–**h**) measured with the setup used in **(i)**. (**k**) Schematic of setup for specular reflectance measurement used in this work. Both the angles of incident and observation light are from the normal direction. (**l**) Normalized specular reflectance spectra of Reflectin and BSA films in (**a**–**h**) measured with the setup used in (**k**). (**m**) Schematic of setup for scattered light measurement. The incident angle is 45°, and the observation angle is 0° (normal direction). (**n**) Normalized scattered reflectance spectra of Reflectin and BSA films in (**a**–**h**) measured with the setup used in (**m**). (**o**) Schematic of setup used for scattered reflectance measurement of R/I and R films in (**c**) and (**f**) under different incident angles. Angle of incident light can be changed (25°, 35°, 45°, 55°, 65°) with the rotation of light source fiber, whereas the detector remains at the normal direction. (**p**) Normalized scattered reflectance spectra of R/I and R films in (**c**) and (**f**) under different incident angles measured with the setup used in (**o**). (**q**) Schematic of setup used for scattered reflectance measurement of R/I and R films in (**c**) and (**f**) under different observation angles. The light source fiber remains at the normal direction, whereas the angle of observation can be changed (25°, 35°, 45°, 55°, 65°) with the rotation of the detector fiber. (**r**) Normalized scattered reflectance spectra of R/I and R films in (**c**) and (**f**) under different observation angles measured with the setup reported in (**q**). The scale bars represent 2 mm.

To identify the origin of the blue coloration observed in reflectin films, different films with various thicknesses were prepared, and their optical characterizations were studied. The SoRef2 protein films with different thicknesses were obtained with different protein concentration (330 mg/ml, 363 mg/ml, and 424 mg/ml) in spin coating experiments. We also deposited the films on cover glasses (Fig. 2a–f) or silicon wafers (Fig. S3a–f). BSA (300 mg/ml) with or without imidazole were used in the control group (Figs 2g,h and S3g,h) to exclude the effect of aromatic molecules used in protein films. Thickness was measured by scanning electron microscope (SEM). Blue coloration occurred in all films of higher-order assembled reflectin treated with imidazole but not in films of self-assembled reflectin without stimulation or the control group, and these results were independent of film thickness (Fig. 2a, 1 μm; Fig. 2b, 2.5 μm; Fig. 2c, 12 μm, Fig. 2d, 0.5 μm; Fig. 2e, 1 μm; Fig. 2f, 5 μm, Fig. 2g, BSA/Imidazole, 4 μm; Fig. 2h, BSA, 4 μm). Films on silicon wafers exhibited similar results (Fig. S3a–h). This observation is somewhat different from previous reports^19,20^, which may due to different sample preparation^26^ and may indicate different protein structures and assembly in different studies.

We measured the transmission and reflection spectra of reflectin films (Fig. 2i,k). Transmission spectra of SoRef2/Imidazole films exhibited an obviously reduced transparence in short wavelengths region (Fig. 2i,j). However, the spontaneously measured reflection spectra did not exhibit evidence of reflecting of light in short wavelengths, indicating that the loss of transmission of this spectral range was not due to the reflection. Instead, we observed reduced intensity of reflectance in thicker SoRef2/Imidazole films in short wavelengths (Fig. 2k,l). The transmission and reflection spectra of SoRef2 and BSA and BSA/Imidazole films were uniform for the entire visible light region.

These experimental findings suggest that the blue coloration of SoRef2/Imidazole films cannot be produced simply by specular reflectance, which led to our hypothesis that a scattering mechanism may be involved in the generation of the blue coloration. We then used a custom system to measure the angle-dependent scattering spectra of the reflectin films. When the detector was positioned at the normal direction with incident light illuminating onto the film with a tilted angle of 45° from it (Fig. 2m), most incident light was specular reflected by the flat surface of films such that detector collected no reflected signal but most of the scattering light. Only SoRef2/Imidazole films exhibited strong light scattering between 450 nm and 500 nm (Fig. 2n). Increasing the thickness facilitated the short-wavelength scattering as shown in intensity increase. In particular, the 12-µm SoRef2/Imidazole film exhibited a significant scattering peak corresponding to a deeper blue appearance, whereas the films without imidazole did not exhibit any scattering signals. Similar scattering spectra were obtained from films prepared with SoRef2/Imidazole on silicon wafers (Fig. S3i), SoRef2/Histamine on glasses (Fig. S3j), SoRef1/Imidazole on glasses (Fig. S3k), SoRef8/Imidazole on glasses (Fig. S3l), and even a single domain (D1) of SoRef2/Imidazole on glasses (Fig. S3m).

As iridescence is common feature of structural color^5^, the angle dependence of optical properties of the SoRef2/Imidazole film (Fig. 2o–r) were measured in two configurations: positioning the detector at the normal direction or light source at the normal direction while changing angles of the other detector (Fig. 2o,q). Although the increased incident or scattering angles correlate to stronger scattering intensity, the similarities in both configurations indicates that scattering arises from structural color (Fig. 2p,r). Unchanged peaks of the scattering spectra suggest that the blue color is angle independent, which is consistent with light scattering assumption.

### Structural analysis of reflectin films

**Figure 3:**
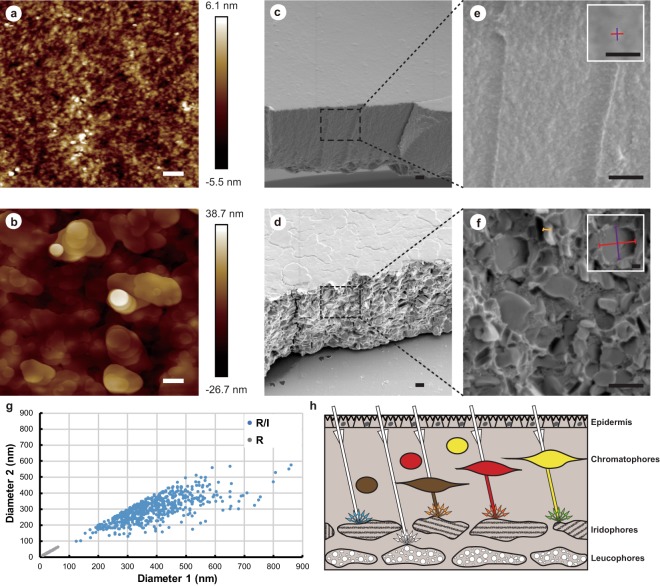
Structural analysis of Reflectin films and model for the color change in cephalopods. (**a**) AFM analysis of surface morphology of colorless Reflectin (R) film. (**b**) AFM analysis of surface morphology of blue Reflectin/Imidazole (R/I) film. (**c**) SEM image of surface morphology of colorless R film. (**d**) SEM image of surface morphology of blue R/I film. (**e**) SEM image of cross-sectional morphology of colorless R film. Diameters of particles on R film (diameter 1 and diameter 2) were measured along two axes separately (shown as red line and purple line), and the higher magnification of this image is presented inside. Particles exhibit a small globular-like shape (Mean diameter 1: 22 nm, mean diameter 2: 22 nm, n = 588). (**f**) SEM image of cross-sectional morphology of blue R/I film. Diameters of large platelet-shaped particles on the cross section of R/I film were measured along the long (shown as red line) and short (shown as purple line) axes separately, and the higher magnification of this image is presented inside. Mean diameter 1: 389 nm, mean diameter 2: 299 nm, n = 581. Thicknesses of large platelet shaped particles on the cross section of R/I film were measured (shown as orange line). The thickness ranged from 40 to 108 nm, and the average thickness is approximately 68 nm. (**g**) Diameters of particles on the cross section of colorless R film and blue R/I film as measured from the SEM image in (**e**,**f**). (**h**) Schematic illustration summarizing the proposed model for the color change mechanism in cephalopods. Structural and mechanistic details elucidated in this manuscript are diagrammed. (See text for details.) The scale bars in (**a**–**f**) represent 500 nm, whereas the scale bars in the higher magnification of (**e**,**f**) shown in white boxes represent 125 nm.

Atomic force microscopy (AFM) and scanning electron microscopy (SEM) were used to study the structural basis of higher-order reflectin-based “cephalopod-blue”. AFM results revealed the surface morphology difference induced by imidazole. SoRef2 film exhibited a relatively smooth surface with a roughness of approximately 11.6 nm (Fig. 3a), whereas the roughness was significantly increased for SoRef2/Imidazole film (65.4 nm, Fig. 3b).

SEM studies of both surface and cross-sectional morphologies further revealed structural morphology differences. Smooth and colorless SoRef2 film was closely packed with smaller globular particles formed by reflectin proteins (Fig. 3c,e), whereas the rough and blue SoRef2/Imidazole film was loosely packed with larger platelet shaped particles formed by reflectin proteins with random orientations (Fig. 3d,f).

Quantitative analysis of particles size distribution provided insights into the origin of “cephalopod-blue”. Particles were treated as approximate ellipsoids, and sizes were measured along two axes (Fig. 3). Through SEM observation of the cross section of the films, the average size of particles in SoRef2/Imidazole film was 389 × 299 nm, and greater than half of the particles exhibited a size ranging from 350–500 nm (Fig. 3g). We also analyzed SoRef2/Histamine film, and the average size was 350 × 260 nm (Fig. S4). Such particle size distribution was comparable with the range of short-wavelength visible light, especially the blue light (450 nm–500 nm), when taking the refractive index into account. Hence, the scattering efficiency of blue light may be effectively enhanced and result in blue coloration in the experiments. Particles distribution in SEM cross section images was counted. Proportion of particles with one diameter in the range of 350 nm~500 nm in SoRef2/Imidazole films was 53.7%. Only 6.4% of particles with one diameter larger than 500 nm. Rest of particles with diameters below 350 nm. The averaged particle sizes in SoRef2 films were about 22 × 22 nm. Two different sizes were selected in FDTD simulation (Fig. S5). The mean size of 389 nm by 299 nm by 68 nm was chosen to present particles in SoRef2/Imidazole films, and 22 nm by 22 nm by 22 nm was selected to approximate particles size in SoRef2 films. As shown in Fig. S5, particle in SoRef2/Imidazole films shows distinct scattering signal in short wavelengths.

Scattering was dependent of particles sizes, which was consistent with our experimental results. Moreover, the average thicknesses of platelet particles in both SoRef2/Imidazole and SoRef2/Histamine films were estimated to be approximately 68 nm and 98 nm, respectively. These numbers are consistent with the plates thickness in iridophores of cephalopods^14^.

### Simulation of cephalopods’ coloration *in vitro*

The remarkable color change ability of cephalopods depends on their special skin components, including chromatophores, iridophores and leucophores^1^. However, different cell types contribute differently to produce the vivid and full spectrum of color in cephalopods. Typically, colors of long wavelengths are produced by three types of chromatophores (red, yellow/orange and brown/black), which act as spectral filters of a specific wavelength^1,33,34^. In contrast, iridophores contribute mostly to short-wavelength colors^11,34,35^. The data presented in this study are consistent with the idea that reflectin produces short-wavelength colors by forming the reflectin films *in vitro* or platelets structures *in vivo*.

The combination of pigmentary color in chromatophores and structural color in iridophores is sufficient to generate more vivid colors with *in vitro* simulations. Briefly, three filters (yellow, red and brown) were chosen based on their transmittance spectrum (Fig. S6a) to mimic the optical properties of three types of chromatophores^33^, and blue higher-order assembled reflectin/Imidazole film was used to simulate the optical features of iridophore. Broadband white light was filtered by three filters separately and illuminated on reflectin/Imidazole film at a titled angle (75°), and scattering light was measured (Fig. S6b). Obvious color shifting was observed (Fig. S6c–h).

The mechanism behind adaptive coloration for cephalopods in a combination of different cell types and integration of both pigmentary color and structural color are suggested previously^34^. By investigation of the connection between structural/assembly status and scattering features of reflectin films, an updated model of this sophisticated coloration system is summarized (Fig. 3h) to emphasize that the blue color is generated by scattering in iridophores, and more vivid colors such as green can be generated by combination of long-wavelength colors in chromatophores and scattered short wavelength color in iridophores. In addition, colors become more significant given the perfect white contrast produced by leucophores.

### Dynamic color change of reflectin films

**Figure 4:**
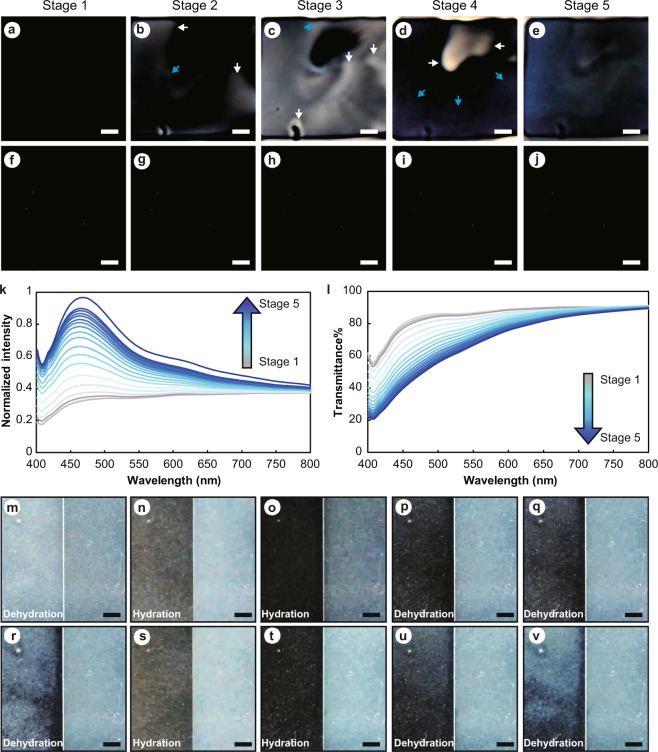
Optical characterization of the dynamic color changing of Reflectin protein. (**a**–**j**) Spin-coated films of Reflectin (SoRef2) incubated with (Reflectin/Imidazole) and without (Reflectin) imidazole. Reflectin/Imidazole film (the upper panel, a–e) exhibited dynamic color changes (stage 1 to stage 5) from colorless to white (indicated by white arrows) followed by blue (indicated by blue arrows) gradually from the edge to the center of the film during the water evaporation process, whereas Reflectin film without aromatic molecules (the lower panel, f–j) remained colorless. (**k**) Normalized scattered reflectance spectra of Reflectin/Imidazole film during the color changing process, corresponding to five stages in (**a**–**e**) measured using the setup reported in Fig. 2m. (**l**) Transmittance spectra of Reflectin/Imidazole film during the color changing process, corresponding to five stages in (**a**–**e**) measured using the setup reported in Fig. 2i. (**m**–**v**) The reversibility of dynamic color change of Reflectin/Imidazole film. Note that the right half of the film was covered with a slide glass to protect from the hydration and dehydration cycles. (**m**) Blue dried Reflectin/Imidazole film. (**n**) Hydration of left half of Reflectin/Imidazole film by water mist spray from an ultrasonic humidifier. (**o**) Left half of Reflectin/Imidazole film turned into colorless gradually along with the hydration process. (**p**) Light blue appeared at the left corner as dehydration started. (**q**) Light blue progressed along with the dehydration of the left half of Reflectin/Imidazole film. (**r**) Left half of Reflectin/Imidazole film turned into blue when dehydration process completed. (**s**) Re-hydration of left half of Reflectin/Imidazole film in (**r**) by water mist spray. (**t**) Left half of Reflectin/Imidazole film turned into colorless again when hydration process completed. (**u**) Light blue appeared at the left top corner as dehydration started. (**v**) Left half of Reflectin/Imidazole film turned into blue again when dehydration process completed. The scale bars represent 2 mm.

Cephalopods exhibit a remarkable ability for dynamic color change, during which reflectin-formed optical nanostructures play a key role^1^. Films formed by reflectin in the presence of aromatic molecules (SoRef2/Imidazole) as described above not only reveal the characteristic “cephalopod-blue” but also present a dynamic structural color change process starting from colorless, transitioning to white and ending with blue (Fig. 4, Movie S1). Briefly, at first, both films (SoRef2/Imidazole and SoRef2) appeared transparent after spin coating (Fig. 4a,f). Soon, white color started appearing from the edge of SoRef2/Imidazole film along with the evaporation of solvent (Fig. 4b), and this broad band scattering gradually expanded from the film edge towards the center and eventually covered the entire surface (Fig. 4c). Later, blue color gradually appeared where the white color faded (Fig. 4d) and eventually covered the entire surface (Fig. 4e). This process has been robustly repeated and similar color change processes were observed with different reflectin proteins (SoRef1, SoRef2 and SoRef8) in the presence of aromatic molecules. Of note, film without aromatic molecules remained colorless during the entire process (Fig. 4f–j).

To quantitatively understand the scattering, we recorded the reflection and transmission spectra during this process. Gradually increased reflection along with the simultaneously decreased transmission illustrated how particles sizes determined the coloration dynamically in SoRef2/imidazole film. Similar to the angle dependence experiments, a peak gradually emerged in the short-wavelength region approximately 450–500 nm (Figs 4k,l, S7). Our observation indicates that the dynamic color change may include three main stages: (1) Higher-order assembly of reflectin wherein diverse sizes can move freely in the solution system, resulting in a colorless film; (2) The movement of reflectin particles slows down as the dehydration proceeds, and a mild increase in the size of reflectin particles and percentage of large particles may occur due to the increased concentration of aromatic molecules and proteins, as indicated by the increased scattering intensity and a slight redshift of the scattering peak. These processes gradually result in color changing from colorless to white to blue. It was assumed that the white color was involved in a different scattering process by particles with various sizes and changing orientations^23,24^; (3) Higher-order assembled structures are uniformly formed and distributed, and the particle growth process is completed in dried film, resulting in blue coloration.

The dynamic color change in cephalopods is fully reversible, so is in our *in vitro* reconstruction system. Water mist spray was used to hydrate and re-hydrate the dried Reflectin/Imidazole film. The initial blue color (Fig. 4m) turned into colorless (Fig. 4n,o) gradually along with hydration, and back to blue again upon dehydration (Fig. 4p–r). This color transition can be reversed repeatedly by hydration and dehydration cycles with the same film (Fig. 4s–v).

## Discussion

To date, we have demonstrated that three different reflectin proteins SoRef2, SoRef1, and SoRef8 as well as a single domain (D1) of reflectin from *Sepia officinalis* exhibit similar features of hierarchical assembly, including self-assembled reflectin bricks and higher-order assembled platelet structure. We reconstructed the dynamic and reversible color change process *in vitro* using spin-coated reflectin films. However, only films formed by higher-order assembled structures of these proteins exhibited dynamic color changes from colorless to white and blue, regardless of film thickness and the type of reflectin protein. Besides, this dynamic blue color change process can be reversed with the hydration and dehydration cycles. The similarity in structure, assembly and optical features of these different reflectin proteins indicates that blue coloration, as well as the dynamic and reversible color change ability may be shared in the reflectin family.

Intriguingly, the blue coloration of higher-order assembled reflectin film resulted in scattering rather than specular reflecting. Reflection and transmission spectra measurements, structural analysis of spin coated films and the particle size distribution results are consistent with this conclusion. In the FDTD simulation, Mie scattering was employed to simulate the scattering phenomenon. A good agreement between experimental results and simulations was obtained. Hence, we hypothesis that the main underlying mechanism of the enhanced blue light scattering is Mie scattering, which happens as light scattered by particles with comparable size to the wavelength of incident light. However, Rayleigh scattering caused by smaller scatters may also occur in the dried SoRef2/Imidazole films due to various sizes of particles. Further investigation is needed. In fact, blue color generated by scattering is not an isolated incident. The iridescent colors in many animals are also generated by scattering. Wavelength-specific forward scattering of light by Bragg-reflective iridocytes has been reported in giant clams^36^, and structural colors of avian feather barbs are produced by light scattering from spongy β-keratin and air nanostructures within the medullary cells of avian feather barb rami^37^. Moreover, structural blue color in the skin of mandrill (*Mandrillus sphinx*) and vervet monkey (*Cercopithecus aethiops*) are observed through coherent scattering from quasi ordered arrays of dermal collagen fibers^2^.

It is worth pointing out that the blue coloration and scattering have been explored with reflectin film by Crookes-Goodson with protein in HFIP and flow coating method previously^31^. However, the structural basis of the blue coloration remains unknown. Here in this study, with protein in native conditions with homogenous structure, we are able to tightly control the protein assembly statuses using aromatic molecules, which allows us to investigate the dynamic color change process from the point of view of structural biology. It is likely that the hierarchical assembly of reflectin protein is the key factor leading to the dynamic structural color change in cephalopods.

The *in vitro* reconstruction experiments by no means to directly suggest what exactly happened *in vivo*, however it may provide biological implications for what could be possible. The combination of pigmentary color produced by chromatophores and reflectin-based structural color produced by iridophores allow cephalopods to produce the widest range of colors and patterns to help them adapt to their marine environments. The blue color with wavelengths ranging from 450 to 500 nm observed in our experiment corresponds well with the blue-green reflections observed in living animals^14^. The structural analysis and optical characterization as well as the *in vitro* reconstruction system we presented here may facilitate our understanding of dynamic color change in cephalopods at the molecular level and serve as an initial step for designing bio-inspired optical materials based on reflectin protein.

## Methods

### Protein expression and purification

The full length reflectin *SoRef2*, *SoRef1* and *SoRef8* genes from cuttlefish *Sepia officinalis* and the protein coding sequences of the reflectin domain 1 (D1, residue 38–85 of *SoRef2* gene) were synthesized and cloned into a customized expression vector with a His-tag fused to the N-terminal driven by a cold-shock promoter (*cspA*) and a *lac* operator^26,38^. All proteins were expressed in *E. coli* strain BL21 (DE3). Bacterial cells were harvested after 20 h of incubation with 20 μM isopropyl β-D-1 thiogalactopyranoside (IPTG) at 288 K and resuspended and sonicated in lysis buffer (20 mM Tris, 150 mM NaCl) on ice.

The purification of SoRef2 and D1 was performed as reported previously^26^. To prepare SoRef1 and SoRef8 proteins, the same purification procedures used for SoRef2 were employed. Briefly, after centrifugation, the precipitates were resuspended and incubated in buffer R (20 mM Tris, 150 mM NaCl, 0.5% Triton X-100, pH 8.0) for 30 min before centrifugation again to remove possible membrane fractions. The pellets were first washed with water thrice (with 5-min incubation for each wash step) and then washed with solubilizing buffer S (20 mM Tris, 150 mM NaCl, 0.05% SDS, pH 8.0) twice (with 30-min incubation for each wash step). The supernatant from previous wash steps was discarded. The majority of the SoRef2 protein was solubilized starting from the third incubation with buffer S and subsequently applied to the Ni-NTA affinity column (QIAGEN, Valencia, California). Ni-NTA affinity chromatography was performed according to standard protocols. The column was washed with washing buffer W (20 mM Tris, 150 mM NaCl, 20 mM imidazole, 0.05% SDS, pH 8.0) for 30 column volumes (CV). Proteins were eluted from the Ni-NTA column using elution buffer E (20 mM Tris, 150 mM NaCl, 300 mM imidazole, 0.05% SDS, pH 8.0) and dialyzed using buffer S (20 mM Tris, 150 mM NaCl, 0.05% SDS, pH 8.0) for the following experiments.

For all SDS-PAGEs, PageRuler Prestained Protein Ladder (Thermo Scientific, Product# 26616) was used as the molecular weight standards marker.

### Reflectin incubated with aromatic molecules

The stock solutions for imidazole (1 M) and histamine (500 mM) were adjusted to pH 8.0. The samples for transmission electron microscopes (TEM) observation were applied to EM grids immediately after adding imidazole or histamine into purified SoRef2, SoRef1, SoRef8 and D1 solution (protein in buffer 20 mM Tris, 150 mM NaCl, 0.05% SDS, pH 8.0). Then, proteins containing imidazole or histamine were concentrated for spin coating. Buffers containing the aromatic molecules imidazole and histamine served as controls. The final concentration of imidazole is 300 mM and histamine is 100 mM.

### EM observation

Negatively stained samples and image acquisition were performed using the standard method as described elsewhere^26,38^. Briefly, 10 µL protein solution was first placed on an EM copper grid. After 1 min, excess protein solution was removed, and 2% (w/v) uranyl acetate solution was used to stain samples. Image acquisition was performed on a Tecnai G2 20 Twin transmission electron microscope (FEI) operated at 120 kV with a nominal magnification of ×50,000, using a dose of ~30 e-Å-2 and a defocus range of −1 to −3 μm. Micrographs were collected by an Ultrascan 4000 charge-coupled device (CCD) camera (Gatan) with a pixel size of 0.427 nm for the specimen.

### Spin coating

Spin coating of reflectin films was performed in cleanroom as reported previously^19^. A drop (approximately 250 μL) was pipetted onto the center of a pre-cleaned glass slide (Fisher Scientific, Pittsburgh, PA) or polished silicon wafer placed on a spin coater, which was operated at constant speed of 2000 rpm for 50 seconds. Different concentrations (330 mg/ml, 363 mg/ml, or 424 mg/ml; measured by nanodrop at 280 nm, corresponding to 2.1 mM, 2.3 mM, or 2.7 mM) of purified SoRef2 protein in buffers containing 300 mM imidazole, 100 mM histamine or no aromatic molecules were used to generate films with different thicknesses. After spin coating, films were dried at room temperature until the color changing process was complete. In addition to SoRef2 protein, SoRef1 protein (416 mg/mL, 3.1 mM), SoRef8 protein (386 mg/mL, 2.3 mM), and D1 protein (410 mg/mL, 14.4 mM) in buffers containing 300 mM imidazole or no aromatic molecules were used to generate corresponding films. BSA (300 mg/mL, 4.5 mM) in buffers containing 300 mM imidazole, 100 mM histamine or no aromatic molecules was used to form films for the control group.

### Photo and movie recording of spin-coated films

Photos were taken, and movies of the dynamic color changing process were recorded using an SLR camera with wild-angle lens under white light with a black or white background.

For Movie S1 (Dynamic color change of Reflectin film): the film on the left was generated by spin coating of 424 mg/ml (A280, 2.7 mM) Reflectin with 300 mM imidazole (Reflectin/Imidazole) on a cover glass, and the film on the right was generated by spin coating of 424 mg/ml (A280, 2.7 mM) Reflectin without aromatic molecules on a cover glass. Two films were placed on a black background immediately after spin coating and the color changing process were recorded.

For Movie S2 (Reversible color change process of Reflectin/Imidazole film): One dried Reflectin/Imidazole film showing blue color was placed against a black background. Note that the right half of this film was used as control and covered with a slide glass to protect from the hydration and dehydration cycles. The hydration of the left half of Reflectin/Imidazole film was achieved by water mist spray from an ultrasonic humidifier. Then, the film was dehydrated and dried at room temperature. This color transition can be reversed repeatedly by hydration and dehydration cycles using the same film and the whole process was recorded.

### Atomic Force Microscopy (AFM) Observation

Atomic force microscopy (AFM) was used to characterize the surface morphology of reflectin films. All images were obtained using a Bruker Bio-Resolve Scanning Probe Microscope (Bruker, Germany) in tapping mode with scan size of 5 μm and scan rate of 0.799 Hz operating in air at room temperature. Images of dried reflectin films on silicon wafers were captured at random locations with surface areas of 5 μm * 5 μm using Nano Scope Analysis (Bruker, Germany) software.

### Scanning electron microscopy (SEM) observation

Scanning electron microscopy (SEM) was used to assess morphological characterization of the surface and cross section of spin-coated reflectin films. Image acquisition was performed on a Quanta FEG 450 scanning electron microscope (FEI) operating at 10 kV using Everhart-Thornley Detector (ETD) in high vacuum mode and nominal magnifications of ×20,006 and ×80,000. Protein films were Au coated (thickness ~ 2 nm) using Ion Sputter Coater MC1000 (Hitachi) before loading into the SEM chamber. Thicknesses of protein films were measured using cross-sectional images.

### Particle analysis

Based on the results of AFM and SEM observations, particles in Reflectin/Imidazole, Reflectin/Histamine or Reflectin films were analyzed. Particle shapes at interfaces were approximated as ellipses. Diameter 1 along the long axis and Diameter 2 along the short axis were measured using ImageJ. In total, 588 particles on the cross section of Reflectin films, 581 particles on the cross section of Reflectin/Imidazole films and 208 particles on the cross section of Reflectin/Histamine films were measured. Particle thicknesses in Reflectin/Imidazole and Reflectin/Histamine films were also measured using ImageJ. Measurements of particle sizes have been done multiple times.

### Optical measurements

A spectrometer (AvaSpec-ULS3648; Avantes, Netherlands) was used for measurements of reflectance and transmittance spectra. A Tungsten Halogen light source of broadband (380–2500 nm) was equipped with a spectrometer. Two fiber cables were selected to conduct the scattering reflectance and transmittance spectra measurements (FC-UV600-2, Avantes). One reflection probe cable (250–2500 nm) with 6 illumination and 1 read fibers was used to measure the specular reflectance spectrum. For transmittance spectrum measurements, a cuvette holder (CUV-UV/VIS, Avantes) was used. The transmittance spectrum reference was measured with a clean cover glass. For specular reflectance measurements, the integrated fiber was used and placed above films at the normal direction.

For scattering reflectance measurements, one illumination fiber was connected with light source and placed above films with a collimating lens (COL-UV/VIS, Avantes) at tilted incident angles or at a normal direction in two different setups, separately. The other detection fiber was connected with a 4X objective (UPlanFLN, NA = 0.13, OLYMPUS) placed at normal direction or at tilted angles. Films are placed flat on a diffuser (400–700 nm, THORLABS) to enhance the collected signals, which did not influence the signal from samples. A homemade rotator was used to change angles of the illuminating fiber or detecting fiber. The reflectance spectrum reference was measured with a clean cover glass calibrated with a white reference tile (WS-2, Avantes).

For simulation of the presence of chromatophores with iridophores, three filters (yellow, red and brown) were selected to simulate chromatophores according to the optical properties of three types of chromatophores^33^. Then, the scattering reflectance of SoRef2/Imidazole film in presence of yellow, red and brown filters were separately measured. A white light source was selected, which was illuminating from a tilted angle (75°) onto film. A different filter (yellow, red or brown) was inserted into the incident light path as layer of chromatophore above the iridophore *in vivo*. Scattering reflectance was measured at the normal direction.

All the spectra were normalized, and smoothed with the standard method^19^.

### FDTD simulations

FDTD simulations were conducted with FDTD Solutions (From Lumerical). Briefly, 3-D Mie scattering using a total-field scattered-field source was employed. A visible light source was selected, and an optical index of 1.59 was used^20^. To maximize the scattering effect, incident light illuminates from the normal direction to the largest face of the particle. Scattering intensities were calculated along two directions: the long axis and short axis. 389 nm by 299 nm by 68 nm was used for simulation of particles in SoRef2/Imidazole films, and 22 nm by 22 nm by 22 nm was used for simulation of particles in SoRef2 films.

## Supplementary information

Supplementary Information

Dynamic color change of Reflectin film

Reversible color change process of Reflectin/Imidazole film

## Data Availability

The datasets generated during and/or analyzed during the current study are available from the corresponding author on reasonable request.
