# Supplementary material for: Reconstruction of Dynamic and Reversible Color Change using Reflectin Protein
Source: Sci Rep. 2019 Mar 26;9:5201. doi: 10.1038/s41598-019-41638-8 (PMC6435677; doi:10.1038/s41598-019-41638-8)
Supplement: Supplementary file 1 — Supplementary Information [file 41598_2019_41638_MOESM1_ESM.pdf]

Supplementary data for

# Reconstruction of Dynamic and Reversible Color Change using Reflectin Protein

Tiantian Cai,[1,4] Kui Han,[2,4] Peilin Yang,[1] Zhou Zhu,[1] Mengcheng Jiang,[2] Yanyi Huang,[2,*] and Can Xie[1,3,*]

*[1]State Key Laboratory of Membrane Biology, Laboratory of Molecular Biophysics, School of Life Sciences, Peking University, Beijing 100871, China*
*[2]Biodynamic Optical Imaging Center (BIOPIC), Beijing Advanced Innovation Center for Genomics (ICG), Peking-Tsinghua Center for Life Sciences, College of Engineering, and School of Life Sciences, Peking University, Beijing, China*
*[3]Beijing Computational Science Research Center, The Chinese Academy of Engineering Physics, Beijing 100084, China*
*[4]These authors contributed equally to this work.*
*[*]To whom correspondence and requests for materials should be addressed. E-mail: Y.H. (yanyi@pku.edu.cn) and C.X. (canxie@pku.edu.cn)*

**Supplementary Movie S1: Dynamic color change of Reflectin film**

The film on the left was generated by spin coating reflectin with imidazole (Reflectin/Imidazole) on a cover glass, and the film on the right was generated by spin coating reflectin protein without aromatic molecules on cover glass. Two films were placed on a black background. Reflectin/Imidazole film exhibited dynamic color change with the evaporation of water. This movie was accelerated 20 times.

**Supplementary Movie S2: Reversible color change of Reflectin/Imidazole film.**

One dried Reflectin/Imidazole film showing blue color was placed against a black background. The right half of this film was used as control and covered with a slide glass to protect from the hydration and dehydration cycles. Obvious color change from blue to colorless can be observed for the left half of Reflectin/Imidazole film upon hydration by water mist spray from an ultrasonic humidifier. Then, the left half of Reflectin/Imidazole film turned into blue again after dehydration, indicating that the blue color change process is reversible. This reversible color transition was repeated twice using the same film, and the whole process was recorded. This movie was accelerated 20 times.

**Supplementary Figures:**

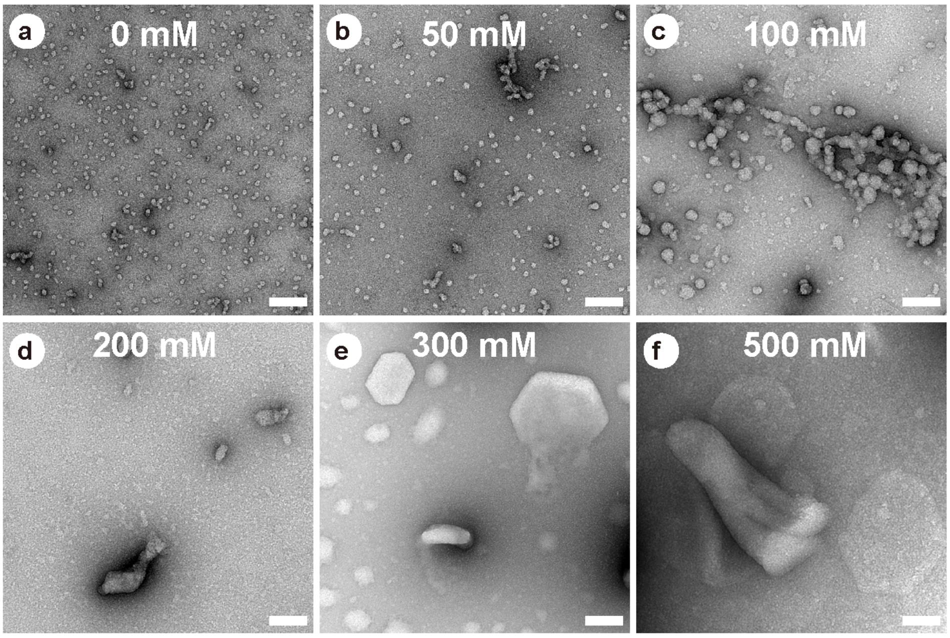


**Supplementary Figure S1. Reflectin protein assembly induced by various concentrations of imidazole:** **a**, Negative-staining EM image of reflectin (SoRef2) particles with no aromatic molecules. **b**, Negative-staining EM image of reflectin (SoRef2) incubated with 50mM imidazole. **c**, Negative-staining EM image of reflectin (SoRef2) incubated with 100mM imidazole. **d**, Negative-staining EM image of reflectin (SoRef2) incubated with 200mM imidazole. **e**, Negative-staining EM image of reflectin (SoRef2) incubated with 300mM imidazole. **f**, Negative-staining EM image of reflectin (SoRef2) incubated with 500mM imidazole. Scale bars, 100nm.

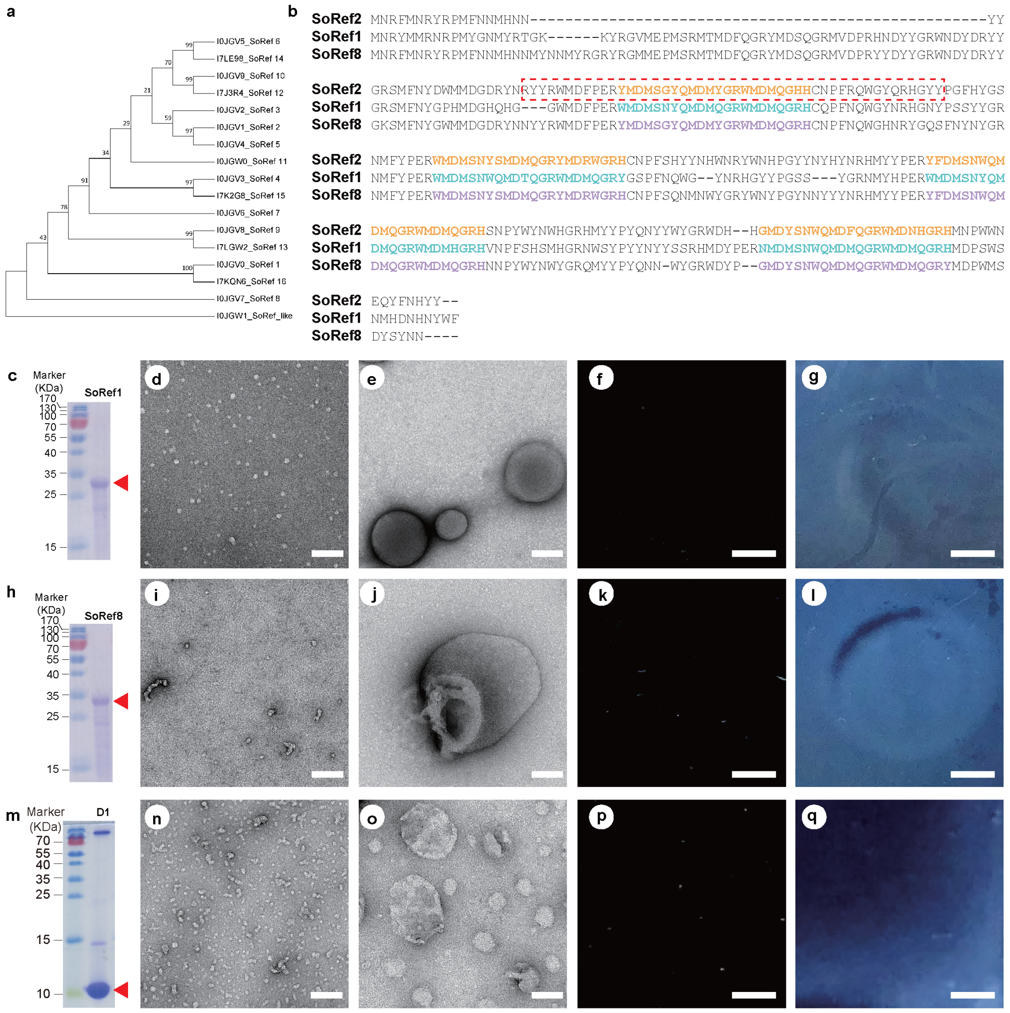


**Supplementary Figure S2. Film formation and coloration of SoRef1 and SoRef8 and a single domain of SoRef2: a**, Phylogenetic tree of reflectin proteins from *Sepia officinalis.* **b**, Sequence alignment of three reflectin proteins (SoRef2, SoRef1 and SoRef8) from *Sepia officinalis*. Core repeats are presented in orange, cyan and purple, separately. A single domain (D1) of SoRef2 is presented inside the red box. **c**, SDS-PAGE of purified SoRef1. **d**, Negative-staining EM image of SoRef1 particles with no aromatic molecules. **e**, Negative-staining EM image of SoRef1 incubated with imidazole (SoRef1/Imidazole). **f**, Film generated by spin coating the SoRef1 sample in **d**. **g**, Film generated by spin- coating the SoRef1/Imidazole sample in **e. h**, SDS-PAGE of purified SoRef8. **i**, Negative-staining EM image of SoRef8 particles with no aromatic molecules. **j**, Negative-staining EM image of SoRef8 incubated with imidazole (SoRef8/Imidazole). **k**, Film generated by spin coating the SoRef8 sample in **i**. **l** Film generated by spin coating the SoRef8/Imidazole sample in **j**. **m**, SDS-PAGE of purified single domain (D1) of SoRef2. **n**, Negative-staining EM image of D1 particles with no aromatic molecules. **o**, Negative-staining EM image of D1 incubated with imidazole (D1/Imidazole). **p**, Film generated by spin coating the D1 sample in **n**. **q**, Film generated by spin coating the D1/Imidazole sample in **o**. The scale bars in **d**, **e**, **i**, **j**, **n**, **o** represent 100 nm. The scale bars in **f**, **g**, **k**, **l**, **p**, **q** represent 2 mm.

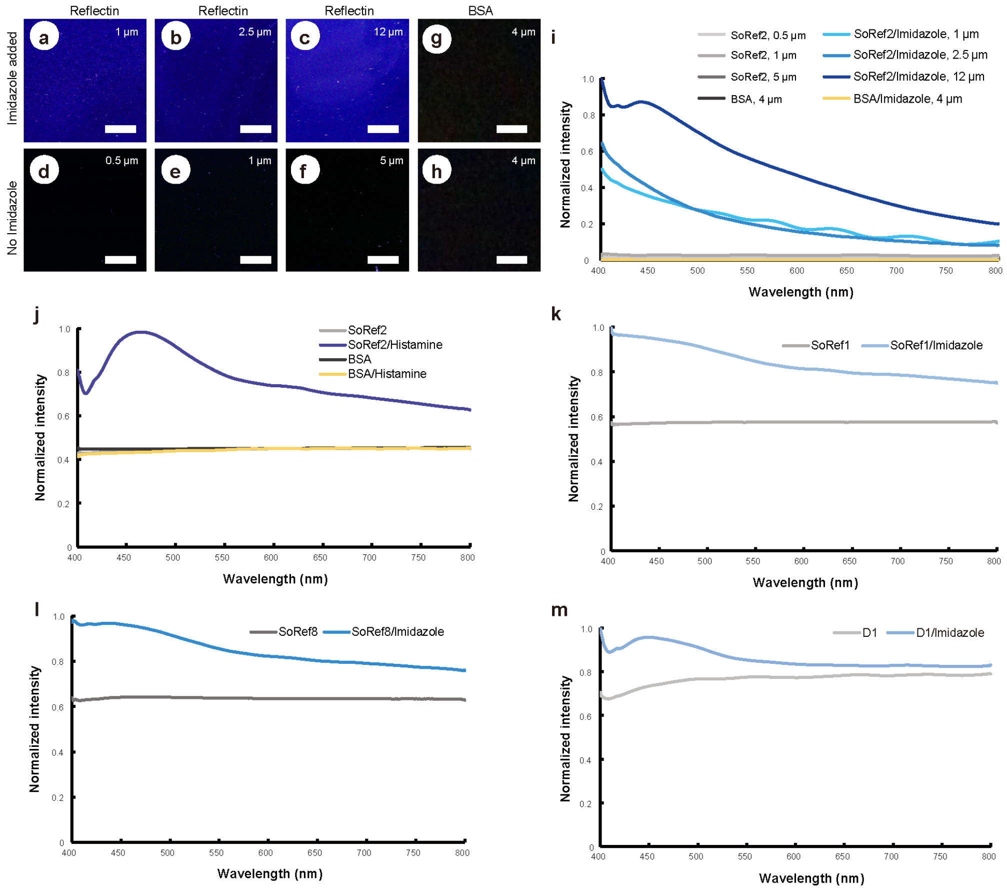


**Supplementary Figure S3. Optical characterization of spin-coated Reflectin proteins and BSA: a-f**, Films of SoRef2 incubated with (SoRef2/Imidazole) and without (SoRef2) imidazole generated by spin coating on silicon wafers. Here, 1 μm (**a**), 2.5 μm (**b**) and 12 μm (**c**) of SoRef2/Imidazole films are blue. In addition, 0.5 μm (**d**), 1 μm (**e**) and 5 μm (**f**) of SoRef2 films are colorless. **g**, **h**, BSA Films of BSA incubated with (**g** is BSA/Imidazole, 4 μm) and without imidazole (**h** is BSA, 4 μm) generated by spin coating on silicon wafers. **i**, Normalized scattered reflectance spectra of SoRef2 and BSA films in **a-h** measured using the setup described in figure 2**m**. **j**, Normalized scattered reflectance spectra of SoRef2 and BSA films with (SoRef2/Histamine, BSA/Histamine) and without histamine (SoRef2, BSA) on glasses measured using the setup in figure 2**m**. **k**, Normalized scattered reflectance spectra of SoRef1 films with (SoRef1/Imidazole) and without imidazole (SoRef1) on glasses measured with the setup described in figure 2**m**. **l**, Normalized scattered reflectance spectra of SoRef8 films with (SoRef8/Imidazole) and without imidazole (SoRef8) on glasses measured with the setup described in figure 2**m**. **m**, Normalized scattered reflectance spectra of D1 films with (D1/Imidazole) and without imidazole (D1) on glasses measured with the setup described in figure 2**m**. The scale bars represent 2 mm.

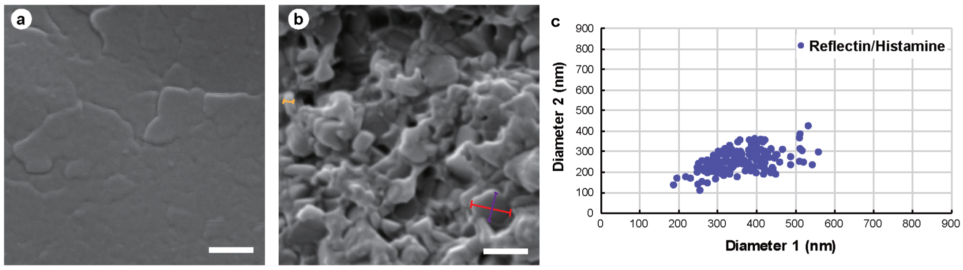


**Supplementary Figure S4. Morphology analysis of Reflectin films: a**, SEM image of surface morphology of blue Reflectin/Histamine film. **b**, SEM image of cross-sectional morphology of blue Reflectin/Histamine film. Diameters of large platelet shaped particles on the cross section of Reflectin/Histamine film (diameter 1 and diameter 2) were measured along long (shown as red line) and short (shown as purple line) axes, separately (mean diameter 1: 350 nm and mean diameter 2: 260 nm, n = 208). Thicknesses of large platelet shaped particles on the cross section of Reflectin/Histamine film were measured (shown as orange line). The values range from 58 to 124 nm, and the average thickness is 97.5 nm. **c**, Diameters of particles on the cross section of blue Reflectin/Histamine film as measured from the SEM image in **b**. The scale bars represent 500 nm.

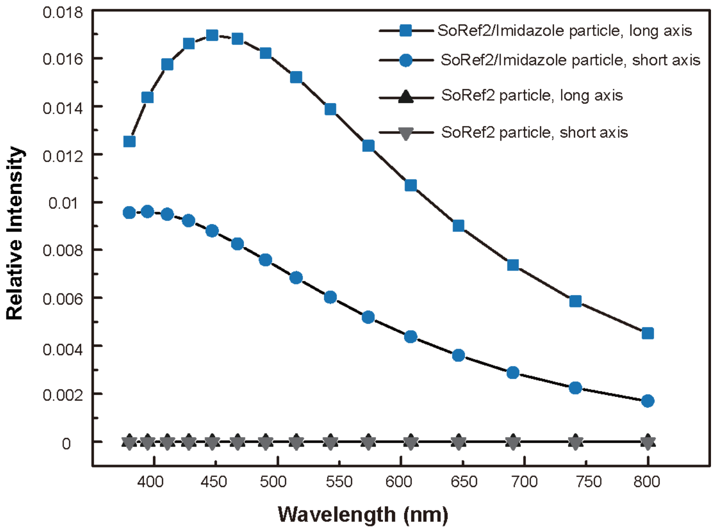


**Supplementary Figure S5. Single particle 3-D Mie scattering simulation results with FDTD:** Ellipsoid shape was used to represent the particles. Two sizes were selected according to SEM observations, 389nm by 299nm by 68nm for SoRef2/Imidazole particles and 22 nm by 22 nm by 22nm for SoRef2 particles. Wavelength dependence of scattering light intensities from different axes was simulated. Results from SoRef2/Imidazole particles are noted in blue, and results from SoRef2 particles are noted in grey.

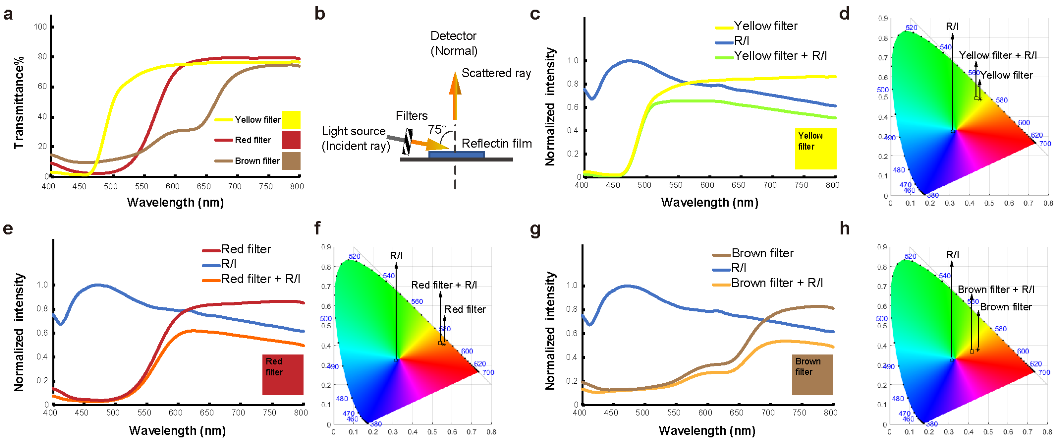

**Supplementary Figure S6. Simulation and reconstruction of color change of cephalopods *in vitro*: a**, Transmittance spectra of yellow, red and brown filters. **b**, Schematic of setup used for scattered reflectance measurement when yellow, red or brown filters are placed above R/I (Reflectin/Imidazole) film. Incident light (75°) was illuminated on R/I film after yellow, red or brown filtering, and scattered light from the R/I film was measured in the normal direction. **c**, Normalized scattered reflectance spectra of the combination of R/I film with a yellow filter (Yellow filter + R/I) measured using the setup described in **b. d**, CIE image indicating the color of filtered light shifted from yellow (Yellow filter) to green (Yellow filter + R/I) after scattering by blue R/I film. **e**, Normalized scattered reflectance spectra of the combination of R/I film with red filter (Red filter + R/I) measured using the setup described in **b. f**, CIE image indicating the color shift to a short wavelength when red-filtered light is scattered by blue R/I film. **g**, Normalized scattered reflectance spectra of the combination of R/I film with brown filter (Brown filter + R /I) measured using the setup described in **b. h**, CIE image indicating the color shift to a short wavelength when brown-filtered light is scattered by blue R/I film.

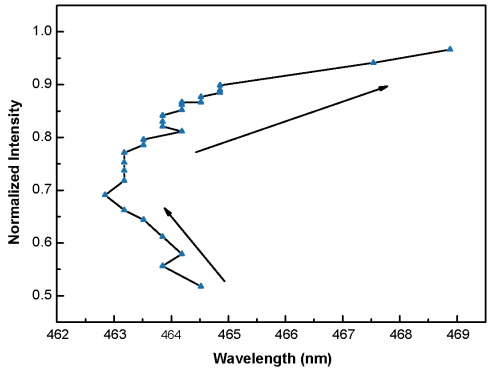


**Supplementary Figure S7. Wavelength dependence of peaks of scattering spectra in short wavelengths:** Peaks are denoted with blue triangles, which are located from 463 to 469 nm. Black arrows represent the progression of the dynamic color change process.
